# Forensically informative nucleotide sequencing (FINS) for the authentication of Chinese medicinal materials

**DOI:** 10.1186/1749-8546-6-42

**Published:** 2011-12-09

**Authors:** Ming Li, Kalin Yan-Bo Zhang, Paul Pui-Hay But, Pang-Chui Shaw

**Affiliations:** 1 State Key Laboratory of Phytochemistry and Plant Resources in West China (CUHK), Institute of Chinese Medicine, The Chinese University of Hong Kong, Shatin, New Territories, Hong Kong SAR, China; 2 School of Chinese Medicine, The University of Hong Kong, Pokfulam, Hong Kong SAR, China; 3 School of Life Sciences, The Chinese University of Hong Kong, Shatin, New Territories, Hong Kong SAR, China

## Abstract

Chinese medicinal materials may be authenticated by molecular identification. As a definitive approach to molecular identification of medicinal materials, forensically informative nucleotide sequencing (FINS) comprises four steps, namely (1) DNA extraction from biological samples, (2) selection and amplification of a specific DNA fragment, (3) determination of the sequence of the amplified DNA fragment and (4) cladistic analysis of the sample DNA sequence against a DNA database. Success of the FINS identification depends on the selection of DNA region and reference species. This article describes the techniques and applications of FINS for authenticating Chinese medicinal materials.

## Background

World Health Organization estimates that 70-80% of the population in the developed countries have used some forms of alternative or complementary medicine [1]. Adulteration and misuse of Chinese medicinal products may be due to (a) accidental substitution due to the similarity of organoleptic characters, (b) inconsistent naming in local areas, (c) intentional substitution of expensive materials by less expensive items and (d) different use of substitutes in local areas. Conventional authentication methods based on organoleptic features and chemical constituents are influenced by various factors such as growing stages, environmental factors and post-harvest processing.

Molecular techniques have been employed to authenticate medicinal materials since the mid 1990s [2]. Molecular techniques, such as DNA fingerprinting, DNA sequencing and DNA microarray, have been applied extensively to authenticate Chinese medicinal materials with a number of these applications having been patented and commercialized [3]. DNA sequencing can retrieve the maximum molecular information from a particular DNA region. Polymorphism of nucleotide sequences provides information to distinguish closely related species from distantly related species and between genuine medicinal materials and adulterants.

Forensically informative nucleotide sequencing (FINS), a technique that combines DNA sequencing and phylogenetic analysis, is used to identify samples based on informative nucleotide sequences. The concept of FINS was first proposed by Bartlett and Davidson in 1992 to identify the origin of animal food products and has since been extensively applied in forensic investigations [4,5]. In the past decade, FINS has been applied to identify and authenticate the Chinese medicinal materials with species-specific DNA regions [6-8].

This article describes the techniques and applications of FINS in authenticating Chinese medicinal materials.

## Performing FINS

**Figure 1:**
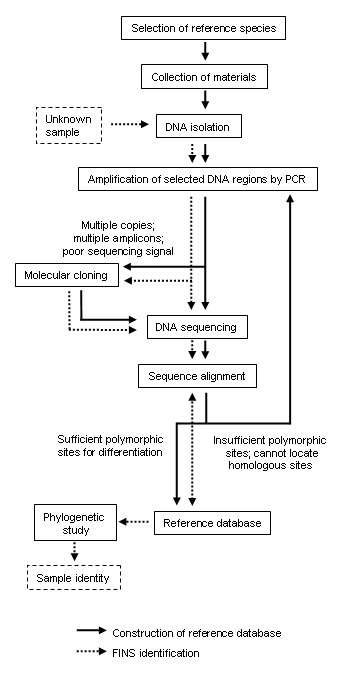
**Schematic diagram showing the workflow of the construction of reference database and FINS identification**.

A defined DNA sequence from examined specimen is obtained and compared with suitable reference sequences from a reliable database using a phylogenetic analysis to identify the tested material [4]. Four basic steps are involved in FINS, namely (1) DNA extraction from biological samples, (2) selection and amplification of a specific DNA fragment, (3) determination of nucleotide sequences and (4) identification using a phylogenetic analysis against a sequence database. Materials used to construct the reference database should be properly identified fresh materials or authentic preserved specimens. The total DNA can be isolated by either DNA extraction or DNA release [9]. A general workflow of FINS is given in Figure 1.

### DNA extraction from biological samples

DNA extraction refers to an invasive method that extracts DNA from tissues and cells via physical disruption and/or chemical fractionation. Cetyl trimethylammonium bromide (CTAB) and phenol/chloroform extraction [10] was employed by a number of commercial kits for DNA extraction. For example, DNA from highly processed Chinese medicinal materials, such as the mule skin extract Asini Corii Colla (Ejiao) [11].

DNA release, a non-invasive method that allows DNA to release from a sample into a solution without destruction, is particularly useful for obtaining DNA from important voucher specimens. DNA release is also used to investigate samples by analyzing the environmental DNA or the preservative, as demonstrated by recent studies of DNA detection from the water in which frogs (*Rana catesbeiana*) live and from worms (*Hypopta agavis*) preserved in 95% ethanol [12,13]. The quantity and quality of the obtained DNA is a major concern with this method. While purification may be achieved by commercially available kits, the yield of DNA is quite minute and should be stored in safe conditions (freezing, cyanide and ethanol), certain chemicals that can damage DNA, such as ethyl acetate or formaldehyde, should be avoided [14].

### Selection and amplification of a specific DNA fragment

Usually, only a small amount of DNA can be extracted or released from highly processed or improperly stored Chinese medicinal materials. Polymerase chain reaction (PCR) can produce a sufficient amount of a specific DNA fragment obtained from a tiny amount of DNA extract. The selection of a DNA region for amplification is one of the crucial factors for FINS because the resolution of FINS depends heavily on the variability and the number of informative sites of the DNA sequences of the tested samples and reference materials. As the evolutionary rates of different DNA regions vary, DNA regions with sufficient variability are essential for providing a high resolution result. Rapidly evolving regions among taxonomic groups can be used for the identification at the genus or species level. Slowly evolving regions among groups can be used to differentiate at the section or family level. An ideal DNA region for identifying Chinese medicinal materials should have high inter-specific variation but low intra-specific variation and have sufficient informative polymorphic sites to allow differential sequence alignment among the samples and the reference species. The evolutionary rate of the same DNA region may vary among animals, plants and fungi. For example, mitochondrial cytochrome c oxidase subunit 1 (*COI*) is suitable for the identification of specific animal species [15]; however, it is not suitable for most plants as few polymorphic sites are found across the 1.4 kb *COI *sequences [16], probably due to the slow mutation rate [17]. Thus, prior knowledge of the evolutionary rates of various DNA regions facilitates the selection of an appropriate DNA region. In the past few years, short DNA sequences for global barcoding of species have been proposed [15]. For example, the DNA barcodes for animals is *COI *and for fungi is ITS; the core DNA barcodes for plants are chloroplast large subunit of ribulose-bisphosphate carboxylase gene (*rbcL*) and chloroplast maturase K coding region (*matK*), while chloroplast *trnH*-*psbA *intergenic spacer (*trnH-psbA*) and nuclear internal transcribed spacer (ITS) are supplementary DNA barcodes for plants [15,18,19]. Recent studies suggested that ITS should be incorporated into the core DNA barcode for seed plants [20-22]. These DNA barcodes have also been commonly applied to identify medicinal materials and should be considered as the primary DNA target region for FINS [23]. Chinese medicinal materials are often dried or processed, which may affect the quality and quantity of the extractable DNA. A shorter DNA region should be considered for samples with degraded DNA. The universal primers for PCR amplification of some commonly used regions in FINS are listed in Table 1.

**Table 1:** Universal primers for PCR amplification of commonly used DNA regions

Region	Primer (5' > 3')		Reference
5S rDNA spacer	S-1	GGATTCGTGCTTGGGCGAGAGTAGTA	[34]
	AS-1	TGCGATCATACCAGCACTAAGGATCC	
12S rDNA	Fwd	CAAACTGGGATTAGATACCCCACTAT	[35]
	Rev	GAGGGTGACGGGCGGTGTGT	
16S rDNA	Fwd	CGCCTGTTTATCAAAAACAT	[36]
	Rev	CTCCGGTTTGAACTCAGATC	
18S rDNA	18SF	CAACCTGGTTGATCCTGCCAGT	[37]
	18SR	CTGATCCTTCTGCACCTTCACCTAC	
*COI*	LCO1490	GGTCAACAAATCATAAAGATATTGG	[15]
	HCO2198	TAAACTTCAGGGTGACCAAAAAATCA	
*Cyt b*	mcb398	TACCATGAGGACAAATATCATTCTG	[38]
	Rev	CCTCCTAGTTTGTTAGGGATTGATCG	
ITS	ITS4	TCCTCCGCTTATTGATATGC	[18]
	ITS5a	CCTTATCATTTAGAGGAAGGAG	
ITS-1	18d	CACACCGCCCGTCGCTCCTACCGA	[39]
	5.8c	TTGCGTTCAAAGACTCGATG	
ITS-2	5.8d	AACCATCGAGTCTTTGAACGCA	[39]
	28cc	ACTCGCCGTTACTAGGGGAA	
*MatK*	3F_KIM f	CGTACAGTACTTTTGTGTTTACGAG	[19]
	1R_KIM r	ACCCAGTCCATCTGGAAATCTTGGTTC	
Mitochondrial control region	L15998	TACCCCAAACTCCCAAAGCTA	[40]
	CSBDH	TGAATTAGGAACCAGATGCCAG	
*TrnH-psbA*	psbA3'f	GTTATGCATGAACGTAATGCTC	[18]
	trnHf	CGCGCATGGTGGATTCACAATCC	
*TrnL-trnF*	Tab C	CGAAATCGGTAGACGCTACG	[41]
	Tab F	ATTTGAACTGGTGACACGAG	
*RbcL*	rbcLa_F	ATGTCACCACAAACAGAGACTAAAGC	[19]
	rbcLa_R	GTAAAATCAAGTCCACCRCG	

### Determination of nucleotide sequences

DNA sequencing is the most direct approach to obtaining maximum genetic information of the amplified DNA regions. With significantly lowered costs and time, DNA sequencing is now routinely used to identify medicinal materials. The amplified and purified DNA fragments may be sequenced directly; however, molecular cloning may be applied in some cases. Cloning is required if (1) some DNA regions (e.g. ITS and 5S rRNA gene spacer) have non-homogenous multiple copies or secondary structures [24,25]; (2) non-specific PCR amplification generates multiple amplicons of similar size; (3) there is simultaneous amplification of DNA from samples and fungal contaminants (e.g. due to improper storage) and (4) there is poly-A/T structure (e.g. in *trnH*-*psbA*) interfering with the DNA sequencing [26].

### Phylogenetic analysis with reference to a sequence database

A sequence database is necessary because a successful application of FINS relies on the comparison of DNA sequences among the samples and reference species. Phylogenetic analyses of many taxa using various DNA regions have been performed, providing useful reference for FINS. Our group has recently constructed an online Medicinal Materials DNA Barcoding Database http://www.cuhk.edu.hk/icm/mmdbd.htm, which contains over 20,000 DNA sequences of 1,300 medicinal species found in the *Pharmacopoeia of the People's Republic of China *and the *United States Pharmacopoeia *[27]. DNA sequences can also be found in the open access NCBI GenBank http://www.ncbi.nlm.nih.gov/genbank, EMBL Nucleotide Sequence Database http://www.ebi.ac.uk/embl as well as the Chloroplast Genome Database http://chloroplast.cbio.psu.edu. With the vast amount of sequence data, it is possible to roughly identify any unknown sample even if the sequence of its source species is not yet available. However, the quality of publicly available DNA sequences could sometimes be incorrect or derived from wrongly identified species [6,28,29]. Generation of tailor-made reference sequences is essential if the concerned reference sequences do not exist and high resolution identification is required.

The original idea of FINS is to perform phylogenetic analysis of unknown samples together with the reference species to trace their source origin [4], which is different from molecular identification based solely on multiple sequence alignment and comparison of polymorphic sites. FINS emphasizes the use of phylogenetic analysis to identify species via phylograms [4]. In general, phylogenetic analysis carefully selects sequence alignment to find the informative homologous sites for subsequent analysis. Phylogenetic trees are then constructed using tree construction methods, such as maximum parsimony (MP), maximum likelihood (ML) and Bayesian analysis, to reflect the evolutionary history of the concerned taxa. Available computer programs for constructing multiple sequence alignment and phylogenetic analysis are Align-M, ClustalW, BioEdit, PAUP and MEGA [30].

To identify medicinal materials, FINS users would rather identify a sample than its phylogenetic relationship with the reference species. The topology of the phylogram is the major concern and the phylogenetic relationship among the reference species is less focused in FINS identification. It was suggested that DNA distance-based methods are preferred to phylogeny-based methods in the application of FINS for identification [31]. The DNA distance-based method provides similarities between the reference and the unknown species whereas the phylogeny-based method explores the evolutionary history of the species. The major difference between these two methods lies in the way that the DNA sequences are analyzed. Phylogenetic relationship analysis, such as maximum parsimony and maximum likelihood, uses a matrix of discrete phylogenetic informative characters or statistical models to infer the optimal phylogenetic trees of selected taxa. Distance-matrix methods, such as unweighed pair-group mean analysis (UPGMA) and neighbor-joining (NJ), calculate the genetic distance from multiple sequence alignments to determine the similarities among reference sequences. A cladogram is then constructed based on the pair-wise distance values to build up the relationship of similarity. The distance-matrix methods are simple to implement and do not invoke any evolutionary indications because similar looking species may not necessarily be phylogenetically related (i.e. convergent evolution).

### Applications of FINS in Chinese medicinal materials

Over 800 medicinal species are officially recorded in the *Pharmacopoeia of the People's Republic of China *[32]. Some of these Chinese medicinal materials are economically important and ecologically valuable, such as Dendrobii Caulis (Shihu), while some others are highly toxic, such as Aristolochiae Fructus (Madouling) and Radix Tripterygii Wilfordii (Leigongteng). FINS is one of the most definitive methods to ensure they are used safely and to protect consumers from adulteration. Over the years, FINS has been used to identify economically important materials and ecologically valuable species, as well as toxic and commonly used Chinese medicinal materials. Examples of the identification of these Chinese medicinal materials using FINS are given in Table 2.

**Table 2:** Representative examples of FINS identification of Chinese medicinal materials

Year	Test material	DNA locus	Major finding	Reference
2002	Shihu samples and *Dendrobium *species	ITS	4 inspected samples of 'Fengdou' were identified	[7]
2003	Rhinoceros horns, *Rhinoceros *species and other mammals	*Cyt b*	2 samples were white rhinoceros and 4 samples were dark rhinoceros	[8]
2004	Snake blood and meat, and 90 snake species	*Cyt b*	6 unknown samples were identified as *Python molurus *and *P. reticulates*	[42]
2005	Chinese sika deer and *Cervus Nippon *subspecies	Mitochondrial control region	2 suspected samples were derived from wild population of *C. Nippon kopschi*	[43]
2007	Fresh and herbal samples of Dangshen	5S rDNA spacer	2 samples of Hong Dangshen were identified as *Codonopsis pilosula *var. *modesta*	[44]
2008	Snake venom	16S rDNA	1 snake venom was identified as derived from *Naja atra*	[45]
2009	Shihu samples and *Dendrobium *species	ITS	Identification of 10 Shihu samples: 6 were *D. officinale*, 1 was *D. nobile *and 3 were *D. denneanum*	[46]
	Shihu samples and *Dendrobium *species	*MatK*	Identification of 4 Shihu samples: 3 were *D. officinale *and 1 were *D. nobile*	[47]
	Shihu samples and *Dendrobium *species	*TrnH-psbA*	Identification of various 'Fengdou' *Dendrobium *species 20 shihu samples	[48]
	Snake venom	16S rDNA	4 snake venom samples were all genuine	[49]
2010	Baihuasheshecao samples and *Hedyotis *species	ITS	4 out of 7 samples were adulterated by *H. corymbosa*	[50]
	Madouling samples, *Aristolochia*, *Cardiocrinum *and *Lilium *species	*TrnH-psbA*, *trnL-trnF*	2 out of 4 Madouling samples from Taiwan and Yunnan were substituted by *C. giganteum *var. *yunnanense*	[51]
2011	Cordyceps samples and related *Cordyceps *species	*EF-1α*, ITS, *nrLSU*, *rpb1*	3 Cordyceps samples were genuine derived from *C. sinensis*, 5 samples were *C. gunni *from China and 1 sample was *C. gunni *from Tasmania	[6]
	Leigongteng samples, *Tripterygium *and *Celastrus *species	5S rDNA spacer, ITS	3 samples of Leigongteng were genuine herb derived from *T. wilfordii*, 2 samples were adulterants derived from *Celastrus *species	[52]

## Requirements for FINS used to authenticate Chinese medicinal materials

FINS has four major requirements on its application in identifying Chinese medicinal materials. Firstly, the success of FINS identification is highly dependent on the quality and amount of the reference sequences [6,28,29]. Confirmation of the authenticity of the reference sequences or generation of tailor-made sequences may be costly and time-consuming. Secondly, FINS requires a reference database to identify any single Chinese medicinal material. Therefore, it is important to select and/or construct various databases with different reference species and different DNA regions to identify a mixture of Chinese medicinal materials. Thirdly, similar to other molecular identification techniques, FINS requires sufficient amount of good quality DNA. Some Chinese medicinal materials are derived from various plant parts with low DNA content and that were highly processed (e.g. by heat, boil or sun-dry). As a result, DNA can be damaged to the point where only very short fragments (< 200 base pair) are left [11,33]. These short DNA fragments may not possess sufficient informative characters for high resolution FINS identification. ITS2 may be a good region for FINS because of its small size (200-300 base pair) and its high variability in plants and animals [20], although molecular cloning is needed to overcome the problem of multiple copies and secondary structure [24,25]. Fourthly, contamination of fungal species is common in Chinese medicinal materials. Specific primers are required for the materials without the amplification of the contaminants when nuclear DNA regions, such as ITS and 5S rRNA gene spacer, are used.

## Conclusion

Using to authenticate genuine medicinal materials, FINS actually traces the identifies of DNA samples at different taxonomic levels. High resolution FINS is expected to be useful in the authentication and quality control of Chinese medicinal materials.

## Abbreviations

*COI*: cytochrome c oxidase subunit 1; CTAB: cetyl trimethylammonium bromide; *cyt b*: cytochrome b gene; *EF-1α*: elongation factor 1α: FINS: forensically informative nucleotide sequencing; ITS: internal transcribed spacer; *matK*: maturase K; ML: maximum likelihood; MP: maximum parsimony; NJ: neighbor-joining; *nrLSU*: nuclear ribosomal large subunit; PCR: polymerase chain reaction; *rbcL*: large subunit of ribulose-bisphosphate carboxylase; *rpb1*: largest subunit of RNA polymerase II; *trnH-psbA*: *trnH-psbA *intergenic spacer; *trnL-trnF*: *trnL-trnF *intergenic spacer; UPGMA: unweighed pair-group mean analysis.

## Competing interests

The authors declare that they have no competing interests.

## Authors' contributions

ML drafted the manuscript. PCS, KYPZ and PPHB critically revised the manuscript. All authors read and approved the final version of the manuscript.

## References

[B1] World Health Organization Traditional Medicine Fact Sheet 2008 N134

[B2] Shaw PC But PPH Authentication of *Panax *species and their adulterants by random-primed polymerase chain reaction Planta Med 1995 61 466 469 10.1055/s-2006-958138 7480209

[B3] Shaw PC Wong KL Chan AW Wong WC But PPH Patent applications for using DNA technologies to authenticate medicinal herbal material Chin Med 2009 4 21 10.1186/1749-8546-4-21 19930671 PMC2791102

[B4] Bartlett SE Davidson WS FINS (forensically informative nucleotide sequencing): a procedure for identifying the animal origin of biological specimens Biotechniques 1992 12 408 411 1571152

[B5] Sahajpal V Goyal SP Identification of a forensic case using microscopy and forensically informative nucleotide sequencing (FINS): A case study of small Indian civet (*Viverricula indica*) Sci Justice 2009 50 94 97 20470742 10.1016/j.scijus.2009.07.002

[B6] Chan WH Ling KH Chiu SW Shaw PC But PPH Molecular Analyses of *Cordyceps gunnii *in China J Food Drug Anal 2011 19 18 25

[B7] Ding XY Wang ZT Xu H Xu LS Zhou KY Database establishment of the whole rDNA ITS region of *Dendrobium *species of "fengdou" and authentication by analysis of their sequences YaoXueXueBao 2002 37 567 573 12914331

[B8] Hsieh HM Huang LH Tsai LC Kuo YC Meng HH Linacre A Lee JC Species identification of rhinoceros horns using the cytochrome b gene Forensic Sci Int 2003 136 1 11 12969614 10.1016/s0379-0738(03)00251-2

[B9] Hajibabaei M deWaard JR Ivanova NV Ratnasingham S Dooh RT Kirk SL Mackie PM Hebert PD Critical factors for assembling a high volume of DNA barcodes Phil Trans R Soc B 2005 360 1959 1967 10.1098/rstb.2005.1727 16214753 PMC1609220

[B10] Kang HW Cho YG Yoon UH Eun MY A rapid DNA extraction method for RFLP and PCR analysis from a single dry seed Plant Mol Bio Rep 1998 16 1 9

[B11] Lv P Zhou X You J Ye BC Zhang Y Extraction of trace amount of severely degraded DNA Z Naturforsch C 2009 64 581 589 19791512 10.1515/znc-2009-7-818

[B12] Ficetola GF Miaud C Pompanon F Taberlet P Species detection using environmental DNA from water samples Bio Letters 2008 4 423 425 10.1098/rsbl.2008.0118 PMC2610135 18400683

[B13] Shokralla S Singer GAC Hajibabaei M Direct PCR amplification and sequencing of specimen's DNA from preservative ethanol Biotechniques 2010 44 305 306 10.2144/000113362 20359306

[B14] Prendini L Hanner R DeSalle R DeSalle R, Giribet G, Wheeler WC Obtaining, storing and archiving specimens and tissue samples for use in molecular studies Techniques in Molecular Evolution and Systematics 2002 Basel: Birkhaeuser Verlag AG 176 248

[B15] Hebert PD Cywinska A Ball SL deWaard JR Biological identifications through DNA barcodes Proc Biol Sci 2003 270 313 321 10.1098/rspb.2002.2218 12614582 PMC1691236

[B16] Cho Y Mower JP Qiu YL Palmer JD Mitochondrial substitution rates are extraordinarily elevated and variable in a genus of flowering plants P Natl Acad Sci USA 2004 101 17741 17746 10.1073/pnas.0408302101 PMC539783 15598738

[B17] Wolfe KH Li WH Sharp PM Rates of nucleotide substitution vary greatly among plant mitochondrial, chloroplast, and nuclear DNAs P Natl Acad Sci USA 1987 84 9054 9058 10.1073/pnas.84.24.9054 PMC299690 3480529

[B18] Kress WJ Wurdack KJ Zimmer EA Weigt LA Janzen DH Use of DNA barcodes to identify flowering plants P Natl Acad Sci USA 2005 102 8369 8374 10.1073/pnas.0503123102 PMC1142120 15928076

[B19] CBOL Plant Working Group A DNA barcode for land plants P Natl Acad Sci USA 2009 106 12794 12797 10.1073/pnas.0905845106 PMC2722355 19666622

[B20] Chen S Yao H Han J Liu C Song J Shi L Zhu Y Ma X Gao T Pang X Luo K Li Y Li X Jia X Lin Y Leon C Validation of the ITS2 region as a novel DNA barcode for identifying medicinal plant species PloS One 2010 5 e8613 10.1371/journal.pone.0008613 20062805 PMC2799520

[B21] China Plant BOL Group Li DZ Gao LM Li HT Wang H Ge XJ Liu JQ Chen ZD Zhou SL Chen SL Yang JB Fu CX Zeng CX Yan HF Zhu YJ Sun YS Chen SY Zhao L Wang K Yang T Duan GW Comparative analysis of a large dataset indicates that internal transcribed spacer (ITS) should be incorporated into the core barcode for seed plants P Natl Acad Sci USA in press 10.1073/pnas.1104551108 PMC3241788 22100737

[B22] Hollingsworth PM Refining the DNA barcode for land plants P Natl Acad Sci USA in press 10.1073/pnas.1116812108 PMC3241790 22109553

[B23] Li M Cao H But PPH Shaw PC Identification of herbal medicinal materials using DNA barcodes J Syst Evol 2011 49 271 283 10.1111/j.1759-6831.2011.00132.x

[B24] Alvarez I Wendel JF Ribosomal ITS sequences and plant phylogenetic inference Mol Phylogenet Evol 2003 29 417 434 10.1016/S1055-7903(03)00208-2 14615184

[B25] Baldwin BG Sanderson MJ Porter JM Wojciechowski MF Campbell CS Donoghue MJ The ITS region of nuclear ribosomal DNA: a valuable source of evidence on angiosperm phylogeny Ann Mo Bot Gard 1995 82 247 277 10.2307/2399880

[B26] Zhu YJ Chen SL Yao H Tan R Song JY Luo K Lu J DNA barcoding the medicinal plants of the genus *Paris* YaoXueXueBao 2010 45 376 382 21351516

[B27] Lou SK Wong KL Li M But PPH Tsui KW Shaw PC Construction of an integrated web medicinal herbal material DNA database BMC Genomics 2010 11 402 10.1186/1471-2164-11-402 20576098 PMC2996930

[B28] Vilgalys R Taxonomic misidentification in public DNA databases New Phytol 2003 160 4 5 10.1046/j.1469-8137.2003.00894.x 33873532

[B29] Nilsson RH Ryberg M Kristiansson E Abarenkov K Larsson KH Koljalg U Taxonomic reliability of DNA sequences in public sequence databases: a fungal perspective PloS One 2006 1 e59 10.1371/journal.pone.0000059 17183689 PMC1762357

[B30] Schmitt I Barker FK Phylogenetic methods in natural product research Nat Prod Rep 2009 26 1585 1602 10.1039/b910458p 19936388

[B31] Forrest AR Carnegie PR Identification of gourmet meat using FINS (forensically informative nucleotide sequencing) Biotechniques 1994 17 24, 26 7946304

[B32] State Pharmacopoeia Commission of People's Republic of China Pharmacopoeia of the People's Republic of China 2010 Beijing: Chemical Industry Press22110548

[B33] Teletchea F Maudet C Hanni C Food and forensic molecular identification: update and challenges Trends Biotechnol 2005 23 359 366 10.1016/j.tibtech.2005.05.006 15927295

[B34] Chen F Chan HY Wong KL Wang J Yu MT But PPH Shaw PC Authentication of *Saussurea lappa*, an endangered medicinal material, by ITS DNA and 5S rRNA sequencing Planta Med 2008 74 889 892 10.1055/s-2008-1074551 18537077

[B35] Girish PS Anjaneyulu ASR Viswas KN Anand M Rajkumar Nb Shivakumar BM Bhaskar S Sequence analysis of mitochondrial 12S rRNA gene can identify meat species Meat Sci 2004 66 551 556 10.1016/S0309-1740(03)00158-X 22060864

[B36] Mitchell SE Cockburn AF Seawright JA The mitochondrial genome of *Anopheles quadrimaculatus *species A: complete nucleotide sequence and gene organization Genome 1993 36 1058 1073 10.1139/g93-141 8112570

[B37] Sogin ML Innis M, Gelfand DH, Sninksky J Amplification of ribosomal RNA genes for molecular evolution studies PCR Protocols: a Guide to Methods and Application 1990 San Diego: Academic Press 307

[B38] Verma SK Singh L Novel universal primers establish identity of an enormous number of animal species for forensic application Molecular Ecology Notes 2003 3 28 31

[B39] White TJ Burns T Lee S Taylor JW Innis MA, Gelfand DH, Sninsky JJ, White TJ Amplification and direct sequencing of fungal ribosomal RNA genes for phylogenetics PCR protocols: A guide to methods and applications 1990 New York: Academic Press 315 322

[B40] Vinas J Tudela S A validated methodology for genetic identification of tuna species (genus *Thunnus*) PloS One 2009 4 e7606 10.1371/journal.pone.0007606 19898615 PMC2764144

[B41] Taberlet P Gielly L Pautou G Bouvet J Universal primers for amplification of three non-coding regions of chloroplast DNA Plant Mol Biol 1991 17 1105 1109 10.1007/BF00037152 1932684

[B42] Wong KL Wang J But PPH Shaw PC Application of cytochrome b DNA sequences for the authentication of endangered snake species Forensic Sci Int 2004 139 49 55 10.1016/j.forsciint.2003.09.015 14687773

[B43] Wu H Wan QH Fang SG Zhang SY Application of mitochondrial DNA sequence analysis in the forensic identification of Chinese sika deer subspecies Forensic Sci Int 2005 148 101 105 10.1016/j.forsciint.2004.04.072 15639603

[B44] Zhang YB Jiang RW Li SL Qiao CF Han QB Xu HX Wong KL But PPH Shaw PC Chemical and molecular characterization of Hong Dangshen, a unique medicinal material for diarrhea in Hong Kong J Chin Pharmaceut Sci 2007 16 202 207

[B45] Chen N Zhao SJ Han LP DNA molecular identification of one snake crude venom used for production ShiZhenGuoYiGuoYao 2008 19 1578 1580

[B46] Liu J He T Chun Z DNA molecular identification of Herba Dendrobii and its adulterant species based on ITS sequence analysis ZhongGuoZhongYaoZaZhi 2009 34 2853 2856 20209944

[B47] Liu J He T Chun Z Analysis and authentication of chloroplast *matK *gene sequences of Herba Dendrobii YaoXueXueBao 2009 44 1051 1055 20055184

[B48] Shao SG Han L Ma YH Shen J Zhang WC Ding XY Analysis and authentication of cpDNA *psbA-trnH *regions of *Dendrobium *species of fengdous YaoXueXueBao 2009 44 1173 1178 20055144

[B49] Chen N Zhao SJ Forensic identification of snake crude venom by mtDNA analysis ShiZhenGuoYiGuoYao 2009 20 2001 2003

[B50] Li M Jiang RW Hon PM Cheng L Li LL Zhou JR Shaw PC But PPH Authentication of the anti-tumor herb Baihuasheshecao with bioactive marker compounds and molecular sequences Food Chem 2010 119 1239 1245 10.1016/j.foodchem.2009.09.013 PMC9799964 36590831

[B51] Li M Ling KH Lam H Shaw PC Cheng L Techen N Khan IA Chang YS But PPH *Cardiocrinum *seeds as a replacement for *Aristolochia *fruits in treating cough J Ethnopharmacol 2010 130 429 432 10.1016/j.jep.2010.04.040 20435131

[B52] Law SK Simmons MP Techen N Khan IA He MF Shaw PC But PPH Molecular analyses of the Chinese herb Leigongteng (*Tripterygium wilfordii *Hook. f.) Phytochemistry 2011 72 21 26 10.1016/j.phytochem.2010.10.015 21094504

